# Catheter indwell time and phlebitis development during peripheral intravenous catheter administration

**Published:** 2014

**Authors:** Kadriye Burcu Pasalioglu, Hatice Kaya

**Affiliations:** 1 Kadriye Burcu Pasalioglu, BSN, Alaca Public Hospital, Çorum/Turkey.; 2 Hatice Kaya, PhD, Associate Professor, Istanbul University, Florence Nightingale Faculty of Nursing Department of Fundamentals of Nursing, Istanbul /Turkey.

**Keywords:** Peripheral venous catheter, Catheter indwell time, Phlebitis, Thrombophlebitis

## Abstract

***Objective:*** Intravenous catheters have been indispensable tools of modern medicine. Although intravenous applications can be used for a multitude of purposes, these applications may cause complications, some of which have serious effects. Of these complications, the most commonly observed is phlebitis. This study was conducted to determine the effect of catheter indwell time on phlebitis development during peripheral intravenous catheter administration.

***Methods:*** This study determined the effect of catheter indwell time on phlebitis development during peripheral intravenous catheter administration. The study included a total of 103 individuals who were administered 439 catheters and satisfied the study enrollment criteria at one infectious diseases clinic in Istanbul/Turkey. Data were compiled from Patient Information Forms, Peripheral Intravenous Catheter and Therapy Information Forms, reported grades based on the Visual Infusion Phlebitis Assessment Scale, and Peripheral Intravenous Catheter Nurse Observation Forms. The data were analyzed using SPSS.

***Results*** *: *The mean patient age was 53.75±15.54 (standard deviation) years, and 59.2% of the study participants were men. Phlebitis was detected in 41.2% of peripheral intravenous catheters, and the rate decreased with increased catheter indwell time. Analyses showed that catheter indwell time, antibiotic usage, sex, and catheterization sites were significantly associated with development of phlebitis.

***Conclusion:*** The results of this study show that catheters can be used for longer periods of time when administered under optimal conditions and with appropriate surveillance.

## INTRODUCTION

Inpatients are exposed to many interventions for diagnosis and treatment. Administration of parenteral medicine is the most commonly used intervention method. More than 80% of inpatients receive intravenous (IV) treatment by insertion of a catheter into the vein.^1^^-^^3^

Administration of parenteral medicine is an integral part of nursing. Nurses administer intravenous liquids or medications prescribed by doctors or clinicians to patients. Nurses also monitor and care for patients.^4^^,^^5^

Intravenous catheters are indispensable tools of modern medicine. Catheters are necessary for administration of liquid treatments and blood and blood products, as well as for parenteral feeding, close monitoring, and infusion of various medicines.

Although IVs have a multitude of uses, they can cause complications that may lead to serious problems such as extravasation, ecchymosis, hematoma, infection, and phlebitis. The most common among these complications is phlebitis.^1^^-^^3^^,^^6^^-^^9^

Phlebitis, defined as inflammation of the venous tunica intima, is a common but avoidable complication in individuals receiving IV treatments. It is generally associated with peripheral venous catheter (PVC) use. Phlebitis is caused by various factors such as wide, thick, and long catheter lumens; catheter material composition; insertion site; number of insertions; indwelling duration; concentration of medicine and solutions; flow rate; contravention of aseptic methods; type of covering used for stabilization; and the rate of infusion set exchange.^2^^,^^4^^,^^10^^-^^15^

There are various and contradictory suggestions for how long a catheter should remain in place. The Infection Control Nurses Association suggests changing the catheters and insertion site every 48–72 hours to minimize the risk of phlebitis. On the other hand, the Centers for Disease Control and Prevention emphasizes that there is no need to change peripheral intravenous catheters in adults more often than 72–96 hours unless there is a risk of phlebitis.^2^^,^^5^^,^^7^^,^^16^^,^^17^

Even though studies have reported an association between phlebitis development and PVC duration, there are no clear guidelines for an optimal or maximum indwell time. In addition, there are insufficient studies on this subject in Turkey. This study was conducted to determine the effect of catheter indwell time on the development of phlebitis.

## METHODS

***Study aim and type of research:*** A cross-sectional study was conducted between May 2011-November 2011 to determine the effect of catheter indwell time on the development of phlebitis during PVC.

***Participants: ***This study included catheters administered to inpatients admitted to one infectious diseases clinic in Istanbul/Turkey. A total of 439 catheters were administered to 103 patients who matched the study criteria, including adults older than 18 years of age willing to cooperate, communicate, and participate in the study and who received IV treatment during hospitalization. Patients in the infectious diseases clinic who were hospitalized for less than one day, such as those who underwent liver biopsies, were excluded from the study. Patients receiving chemotherapy were also excluded.

***Data collection tools:*** Data were collected using number of forms and assessment systems, including a Patient Information Form, Information Form on Peripheral Venous Catheter and Treatment, Visual Infusion Phlebitis Assessment Scale (VIPAS) Staging Key for Peripheral IVs, and the Peripheral Venous Catheter Nurse Observation Form, as described below. The Patient Information Form included demographic features such as age, sex, and medical diagnosis.

The authors developed the Information Form on Peripheral Venous Catheter and Treatment after review of relevant literature. The form collected data on the number of catheters; anatomical site and frequency of catheter administration per site; use of antibiotics and fluids; duration of catheter stay in the vein; phlebitis development; phlebitis level; and whether the catheter has instruments like triple taps, vein valve, and Dosi-flow. 

The VIPAS Staging Key for Peripheral IVs, developed by Alyce Schultz and Paulette Gallant and published by the Intravenous Nurses Society in 2006, is still valid and widely used. It includes an evaluation of potential risks during catheterization, defines and describes phlebitis grades, and offers recommendations for each grade.^18^

***VIPAS classifies phlebitis into 5 grades:*** Grade 1 has none of the typical symptoms of phlebitis such as pain, rash, and edema. Only catheter observation is recommended. Grade 2 describes the early symptoms of phlebitis, such as pain upon palpation and a rash less than 2.5 cm in diameter around the catheter site. VIPAS suggests catheter replacement. Grade 3 phlebitis refers to mid-level disease: IV site rashes are between 2.5 and 5 cm, and palpation of the IV site reveals pain and rigidity. Treatment should be considered after catheter replacement and doctor notification. Grade 4 phlebitis is advanced phlebitis or the onset of thrombophlebitis. At this level, there is a rash larger than 5 cm around the IV site and pain and rigidity upon palpation. VIPAS recommends catheter replacement, doctor consultation, and consideration of treatment. Finally, grade 5 phlebitis describes advanced thrombophlebitis. This grade includes grade 4 symptoms as well as purulent drainage. Recommendations include treatment consideration after catheter replacement and doctor notification. 

The Peripheral Venous Catheter Nurse Observation Form was developed by the authors in order to record observations. Catheters observed through VIPAS were checked every 8 hours, and observations recorded on the form.

***Ethical İssues:*** In order to use VIPAS in this study, written consent was obtained from Alyce Schultz and Paulette Gallant. Before collecting data, written consent from both the Directorate of Nursing Services in the hospital where research was carried out and the Istanbul University Cerrahpasa Faculty of Medicine Clinical Research Ethics Committee were obtained. Individuals enrolled in the study were informed about its aim and risks. The expectations of patients were expressed and informed consent was obtained through the principle of willingness and voluntariness.

***Data Analysis:*** Data were analyzed using SPSS version 11.0 (SPSS, Inc., Chicago, IL, USA). The incidence of phlebitis was compared to individuals’ sex and age groups, duration of venous catheter, whether the patient received any medicine or fluid, catheter size, and site of catheterization. The chi-square test was used to test the significance of the associations. Seven variables determined to be significantly associated with phlebitis development in PVC were evaluated using logistic regression analysis. Incidence of phlebitis was treated as the dependent variable, and patient sex, number of catheterizations, catheter sites, use of antibiotics, number and types of IV fluids administered, and catheter duration were analyzed as independent variables by modeling using the “Enter” method.

## RESULTS

***Patient demographics and catheter and treatment characteristics: *** The mean participant age was 53.75±15.54 (range, 21–90) years; 16.5% were between 20 and 40 years of age (n=17), 59.2% were 41–64 years of age (n=61), and 24.3% were more than 65 years of age (n=25). Men constituted 59.2% (n=61) of the study population. (Table-I)

22 Fr catheters comprised 80.6% (n=354) of the catheters included in the study, 11.4% (n=50) were number 24, and 8% (n=35) were 20 Fr. Vein valves were used in 70.8% of catheters (n=311), triple taps were used in 5% (n=22) and Dosi-flow was used in 1.8% (n=8) of catheters in the study population. Forearms were the most common catheter site, with 59.9% (n=263) of catheters, followed by 25.1% (n=110) to the outer hand, 9.3% (n=41) to the inner elbow, 3.6% (n=16) to the upper arm, and 2.1% (n=9) on the foot. First-time catheter site interventions comprised 11.8% (n=52) of total interventions, and 88.2% (n= 387) were repeat interventions .

Antibiotics were administered in 78.4% (n=344) of catheters, while 21.6% of catheters were not used for antibiotic treatment (n=95).

***PVC Duration and Development of Phlebitis:*** The data showed that 42.4% (n=186) of catheters included in the study were attached to the vein for less than 48 hours, 31.9% (n=140) for 49–96 hours, and 25.7% (n=113) for 97–120 hours. In addition, 41.2% (n= 181) of patients developed phlebitis (grades 2 and 3) and 35.8% (n=157) of catheters did not work properly. Treatment ended and catheters were removed in 3.6% (n=16) of patients, and 19.4% (n=85) were removed after more than 120 hours. No patients developed grade 4 or 5 phlebitis (Table-II).

***Comparison of PVC Duration and Development of Phlebitis: ***Chi-square analysis of catheter site phlebitis due to PVC duration showed a statistically significant difference between groups (p<0.001). More advanced analysis showed that this difference was between all dual groups (p<0.001), the phlebitis rate in catheterizations lasting less than 48 hours was significantly higher than the other groups, the phlebitis rate decreased in the catheters that remained in place for between 49 and 96 hours, and that the rate was the lowest in catheters that remained in place for 97–120 hours. 

The risk for phlebitis in patients with catheters attached for less than 48 hours was 5.8 times that in patients with catheters that stayed for 97–120 hours (p=0.000), and patients with catheters that remained in place for 49–96 hours were 2.8 times more likely to develop phlebitis than those with catheters that remained in place for 97–120 hours (p=0.002, Table-III).

***The Effect of Seven Variables on Phlebitis Development due to PVC Administration:*** Analysis of the effects of 7 variables associated with phlebitis showed that catheter indwell time has the greatest effect (p=0.000), followed by use of antibiotics (p=0.002), patient sex (p=0.007), and catheterization site (p=0.034). These variables are listed according to the Wald test results for the statistical significance of the regression coefficients. Cannula number/size (p=0.140) and the number of fluids injected into the vein (p=0.335) were not significantly associated with phlebitis development (Table-IV).

Approximately 41.2% of PVCs in this study resulted in phlebitis; the majority of cases (90.1%) were grade 2. Patients with catheters attached for less than 48 hours and for 49–96 hours had a 5.8- and 2.8-fold increased risk, respectively, of developing phlebitis compared to patients with catheters inserted for 97–120 hours. Female patients were 1.9 times more likely to develop phlebitis than male patients.

Patients administered antibiotics through catheters had 2.4 times the risk of developing phlebitis. Patients who received total and other parenteral nutrition (hepatamine, 10% dextrose) as an IV fluid developed phlebitis 3 times more often than patients who were administered 5% dextrose, isolyte, 0.9% NaCl, blood and blood products, or no fluid.

## DISCUSSION

Peripheral intravenous catheters, used for both treatment and care, make it possible to administer fluid-electrolytes, blood and blood products, medicine, and parenteral nutrition, and to access veins for hemodynamic observation. Phlebitis, the most common complication, affects 75% of inpatients. Phlebitis is a significant clinical problem. It negatively affects the comfort of the patient, the duration of catheter use, the hospitalization period, and treatment costs.

This descriptive cross-sectional study analyzed a total of 103 patients administered 439 catheters to determine the effect of PVC duration on the development of phlebitis. Factors such as age, sex, number of catheterizations, catheter attachment sites, frequency of interventions, and administration of antibiotics and parenteral fluids through catheters can affect the development of phlebitis. Therefore, these variables are included in this study.

PVCs developed phlebitis in 41.2% of subjects. Significant differences in phlebitis stage distribution were observed between groups. Advanced analyses showed the highest incidence rate in catheters inserted for 48 hours or less, with a decreased rate in catheters inserted for 49–96 hours, and the lowest rate in catheters remaining in place for 97–120 hours.

**Table-I:** Demographic, catheter and treatment features of the individuals having PVC

***Features***	***n***	*** %***
Age		
20-40	17	16.5
41-64	61	59.2
65 +	25	24.3
Mean Age 53.75±15.54 (Min 21-Max 90)		
Sex		
Male	61	59.2
Female	42	40.8
Catheter Number		
24 Fr	35	8.0
22 Fr	354	80.6
20 Fr	50	11.4
Triple tap		
Present	22	5.0
Absent	417	95.0
Vein Valve		
Present	311	70.8
Absent	128	29.2
Dosiflow		
Present	8	1.8
Absent	431	98.2
Anatomical Site of PVC		
Outer Hand	110	25.1
Forearm	263	59.9
Inner elbow	41	9.3
Upper Arm	16	3.6
On the foot	9	2.1
The frequency of Interventions on the site		
For the first time	52	11.8
Used repeatedly	387	88.2
Any Antibiotics Given		
Yes	344	78.4
No	95	21.6

**Table-II:** Duration of PVC stay and the state of phlebitis development

***Features***	***n***	***%***
Duration of Catheter stay in vein		
48 hours	186	42.4
49-96 hours	140	31.9
97-120 hours	113	25.7
The State of Phlebitis Development		
Phlebitis Developed	181	41.2
Phlebitis Not Developed	258	58.8
The Level of Phlebitis		
Grade 1: No Symptom of Phlebitis	258	58.8
Grade 2: Early Symptoms of Phlebitis	163	90.1
Grade 3: Mid-phases of Phlebitis	18	9.9
The Cause of Catheter Removal		
Phlebitis Developed	181	41.2
It didn’t work	157	35.8
The treatment is over	16	3.6
It exceeded 120 hours	85	19.4

**Table-III:** The association between phlebitis development state and duration of PVC stay in vein

***Catheter Duration of Stay in Vein***	***Phlebitis Development***				***χ2***	***P***
	***Present***		***Absent***			
	***n***	***%***	***n***	***%***		
48 Hours	111	59.7	75	40.3	56.940	0.000
49-96 Hours	52	37.1	88	62.9	(sd: 2)	
97-120 Hours	18	15.9	95	84.1		

**Table-IV:** The effect of seven variables on phlebitis development due to PVC Administrations: Logistic Regression Analysis Results (N= 439).

***Variables***	***B***	***Wald***	***Sd***	***P***	***Exp*** ***(B)***	***% 95 Reliability =C.I. EXP(B)***	
1.Sex (Male:0/Female:1)	0.629	7.163	1	0.007	1.876	1.183	2.973
2.Catheter no		3.938	2	0.140			
Catheter no 1 (no20:0 / no 22:1)	0.581	1.358	1	0.244	1.788	0.673	4.753
Catheter no 2 (no20:0 / no 24:1)	-0.033	0.003	1	0.956	0.968	0.302	3.103
3. Catheter Site		8.683	3	0.034			
Site 1 (upper hand-foot:0/ forearm:1)	0.775	8.321	1	0.004	2.171	1.282	3.678
Site 2 (upper hand-foot:0/upper arm:1)	0.401	0.392	1	0.531	1.493	0.426	5.239
Site 3 (upper hand-foot:0/inner elbow:1)	0.367	0.759	1	0.384	1.443	0.632	3.296
4. Use of antibiotics (no: 0 / yes:1)	0.892	9.228	1	0.002	2.440	1.372	4.338
5.No. of fluids (no fluid-1 fluid: 0 /2fluids:1)	0.368	0.928	1	0.335	1.445	0.683	3.058
6.Type of Fluid(: 0, TPN or other: 1)	1.096	6.452	1	0.011	2.993	1.285	6.975
7. Duration of catheter		32.052	2	0.000			
Duration 1 (97-120h:0 / 48h:1)	1.758	30.915	1	0.000	5.802	3.122	10.783
Duration 2 (97-120h:0/ 49-96h:1)	1.027	9.937	1	0.002	2.792	1.475	5.288
(Fixed)	-3.583	34.349	1	0.000	0.028		

Dependent Variable: state of phlebitis development

χ2= 103.729 sd=11 p= 0.000 (Model adaptable)

In a booklet published in 2011, the Centers for Disease Control and Prevention called attention to 2 points related to PVC duration: it is not necessary to change PVCs more frequently than 72–96 hours unless there is a risk of phlebitis, and that there is no recommendation to replace PVCs except for clinical indications.^17^

Other studies have also reported on the relationship between IV catheterization and phlebitis: Lai (1998) detected a phlebitis rate of 3.2 after 24 hours of catheter indwell time, 3.5 at 48 hours, 3.3 at 72 hours, and 2.6 after 96 hours, asserted that IVs can be used safely in these periods. Uslusoy (2006), however, found that phlebitis rates were lowest at 0–24 hours. Maki and Ringer (1991) detected phlebitis risk after 48 hours. Tohid et al. (2005) established a 3% rate of phlebitis during 96 hours of catheterization.^2^^,^^19^^,^^20^

Since there is lack of information in the literature about catheter indwell time, duration of PVC indwell time is also unknown. The results of our study support 96 hours of indwell time; this study is also a resource for related issues.

We conclude that it is possible to increase catheter indwell time by close observation of surgical asepsis principles and by taking measures to prevent development of phlebitis.

## Notes

***Conflicting of interest:*** None.

## Notes

***Acknowledgements:*** We are grateful to the Istanbul University Scientific Research Projects Unit for supporting the study (Project no: 15505).
